# Is pyroptosis a brake or an accelerator in the fate of the tumor?

**DOI:** 10.1038/s41419-025-07866-9

**Published:** 2025-07-28

**Authors:** Yixuan Hou, Wei Li, Jiaying Yang, Haoyang Yu, Congcong Wang, Yanru Li, Shuang Lv, Ling Zhang

**Affiliations:** College of Basic Medical Sciences, The Medical Basic Research Innovation Center of Airway Disease in North China, Key Laboratory of Pathobiology, Ministry of Education, Jilin University, Changchun, China

**Keywords:** Cell death, Tumour immunology

## Abstract

Cancer currently stands as a formidable challenge confronting humanity. Patients afflicted with malignancies typically endure diminished survival rates and compromised quality of life. Consequently, the paramount objective of oncological research lies in redefining cancer from a terminal diagnosis to a clinically manageable condition, thereby realizing this transformative medical paradigm. The development of multiple innovative therapeutic strategies may enhance the antitumor immune response. Notably, pyroptosis is considered both a form of programmed cell death and a highly inflammatory type of immunogenic cell death. Its activation may accelerate cancer cell death and exert antitumor effects as a promising scenario to reverse immunosuppression. This article elaborates on the following three main aspects: the molecular mechanisms underlying pyroptosis and the development of promising therapeutics that target pyroptosis, the relationship between pyroptosis, inflammation, and tumors, and the application of nano-targeting materials in tumor treatments. These interactive therapeutic patterns may catalyze a paradigm shift in oncology. In this review, we probe the synergistic effects of pyroptosis and nanomaterials, forecast achievements in the field of tumor treatments, detail optimized therapeutic strategies, and shed promising light on the potential for the improvement and development of clinical drugs based on pyroptosis.

## Facts

- Multiple innovative therapeutic strategies are needed to improve antitumor immunotherapy.
- A keen understanding of the process of cell death is needed to develop preventative and targeted therapies for cancer.
- Pyroptosis is a type of programmed cell death, and triggering pyroptosis may activate an immune response to tumors.
- Combining nanotherapies with pyroptosis has the potential to successfully eradicate tumor cells and potentially improve the treatment of cancer.

## Introduction

Cancer, a serious priority public health issue, accounts for a large proportion of the primary causes of death worldwide because of its high incidence and mortality [1]. This has resulted in an increased burden on families and society and adverse effects on the well-being of patients. In previous studies detailing the treatments of cancers, the prevailing approach was that promoting apoptosis was one of the most effective treatment methods available. However, this approach to anti-tumor treatment has flaws, as various cancers can be resistant to chemotherapy [2]. Flaws in apoptosis often cause failed anti-tumor treatments [3]. Cell death, i.e., pyroptosis, has been a recent focus among researchers because of the association with innate immunity and diseases [4]. Pyroptosis may be involved in antitumor immune response and has the potential to reverse immunosuppression [5]. Various strategies have been explored to harness pyroptosis as an anti-tumor agent. Recent studies demonstrated that the induction of pyroptosis in tumor cells facilitates anti-tumor activity. The studies underlined gasdermins (GSDMs) as potential new targets for antitumor activity [6]. Pyroptosis triggers a robust pro-inflammatory response, starkly contrasting with the immunologically silent nature of apoptosis [7] and the moderately pro-inflammatory nature of necrosis [8]. Pyroptosis uniquely drives inflammation by amplifying inflammatory cascades through its distinct molecular mechanism. It is characterized by gasdermin-mediated pore formation in the plasma membrane, followed by osmotic imbalance and cellular rupture. These events lead to the release of intracellular cytokines (e.g., IL-1β, IL-18) and pro-inflammatory mediators, which recruit immune cells, activate systemic immunity for the elimination of intracellular pathogens (e.g., viruses), and establish a self-reinforcing inflammatory feedback loop. The induction of mixed cell death types may also provide options to overcome treatment-resistant cancers [9]. Lu et al. showed that molecular targeted therapies elicited concurrent apoptotic and gasdermin E (GSDME)-dependent pyroptotic tumor cell death [10]. This review focuses on pyroptosis.

Researchers have increased knowledge and gained consensus on the accepted definitions and descriptions of pyroptosis. Current reported manifestations of pyroptosis include cell swelling, membrane rupture, and cellular immunostimulatory content release [11].

However, pyroptosis is considered both a programmed cell death [12] and an immunogenic cell death [9], which rises above the immunogenically inert level and has encouraging potential for anti-tumor immunotherapy. The biological effects of pyroptosis are significant in the development and prognosis of tumors, showing concrete manifestations in two aspects. Pyroptosis in tumor cells can cause forceful degradation of tumors, but it can also foster the development of the tumor microenvironment (TME).

Pyroptosis is capable of regulating cell death in a proinflammatory manner, which relies on the caspase and GSDM families [9]. This regulation process is accompanied by inflammatory and immune responses [13]. The activation of inflammasomes initiates the operation of the pyroptosis pathway [14]. Inflammasomes are multiprotein complexes within the innate immune system that function as pattern-recognition receptors. They orchestrate caspase-1 activation and drive inflammatory responses upon detecting pathogen-associated molecular patterns (PAMPs) or host-derived danger signals, such as damage-associated molecular patterns (DAMPs) [2, 15, 16]. Therefore, the strategic position of inflammasomes must be envisaged. The subtle composition and specific functions of inflammasomes are detailed in this review. Pyroptosis is also highly immunogenic owing to its proinflammatory function and the release of DAMPs and cytokines. Activating GSDMs can boost the stimulation of both innate and adaptive tumor immunity [6, 17]. This activation can enhance the immune system’s ability to battle cancer by facilitating a highly efficient anti-tumor immune response. Song et al. reported that two-dimensional NiCoOx nanosheets promote intense cell pyroptosis and can be utilized in ultrasound-augmented catalytic tumor nanotherapy [18]. In addition, research has shown that anti-tumor treatments related to nanomaterials can be highly effective. A detailed description of nanomaterials is covered in part five of this article.

This article reviews the sophisticated molecular mechanisms of pyroptosis and illustrates the potential of pyroptosis as a promising mechanism for effective anti-tumor activity. We outline the relationship between pyroptosis, inflammation, and tumors. Finally, we discuss therapeutic approaches for using nanomaterials as delivery carriers. According to multiple studies, using pyroptosis and nanomaterials collectively is expected to lead to significant achievements in tumor treatments and may offer promising survival advantages to patients with cancer.

## Review

### Part 1: The history and latest definition of pyroptosis

**Fig. 1:**
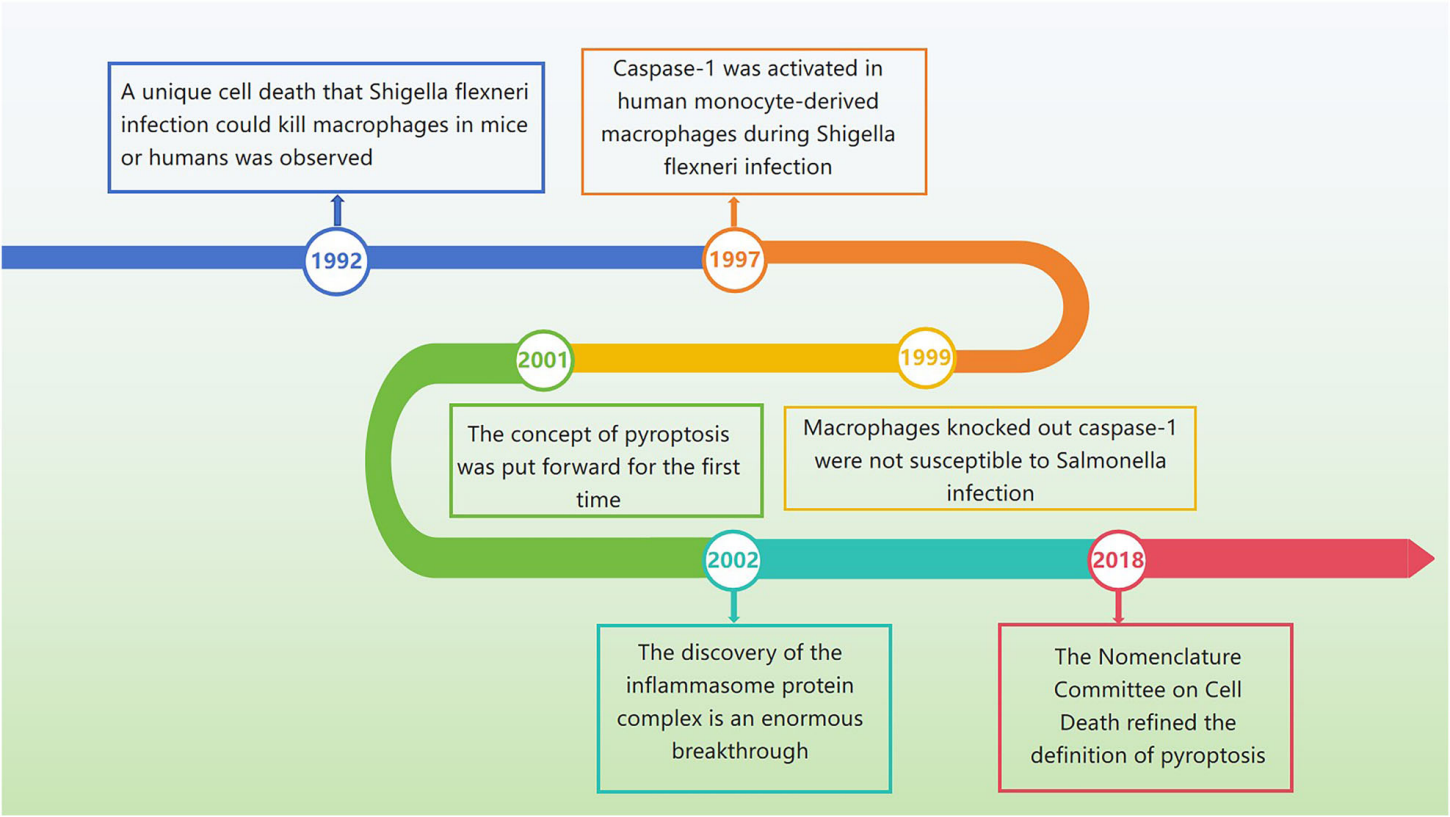
Important moments in the history of pyroptosis. The timeline records the development history of pyroptosis: from the first discovery of pyroptosis to the definition of pyroptosis.

The term pyroptosis is derived from the Greek roots ‘pyro’ and ‘ptosis’, which are defined as ‘fire’ and ‘falling’, respectively. Researching the history of the term can help elucidate the concept of pyroptosis (Fig. 1). In 1992 and 1996, respectively, Zychlinsky et al. observed a unique cell death scenario in which *Shigella flexneri* infection killed macrophages in mice or humans [19]. Subsequently, other researchers discovered that caspase-1 is activated in human monocyte-derived macrophages during *Shigella flexneri* infection [20–22]. According to Hersh et al., macrophages lacking caspase-1 are not susceptible to *Salmonella* infection and are resistant to death [23]. Caspases, closely associated with apoptosis, are aspartate-specific cysteine proteases and members of the interleukin-1β-converting enzyme family. Previously, caspase dependence was regarded as a hallmark of apoptotic cell death [24]. Hence, a relatively prevailing viewpoint has been that caspase-1 activity is necessary for apoptosis to occur in response to *Salmonella* infection [23].

Pyroptosis, a type of proinflammatory programmed cell death, is similar to other forms of cell death, such as necrosis and apoptosis [25]. However, pyroptosis has unique properties that distinguish it from these other forms. Some characteristics are universal to both pyroptosis and apoptosis. Examples may include DNA damage, chromatin condensation, and dependence on caspases [26]. However, pyroptosis has a proinflammatory nature and causes ‘cracks’ in the cell membrane, whereas apoptosis is not proinflammatory and maintains the membrane integrity. Although necroptosis also elicits disruption of the plasma membrane, membrane rupture is more extensive than that of the plasma membrane leakage seen with pyroptosis [27]. Numerous established experimental approaches enable the precise discrimination between apoptosis and pyroptosis. Firstly, morphological analysis, such as transmission electron microscopy (TEM) [28, 29], reveals distinct ultrastructural features: pyroptotic cells exhibit early-stage swelling (a “ballooning effect”) with plasma membrane pore formation (mediated by gasdermin proteins) and late-stage membrane rupture accompanied by cytoplasmic bubble-like protrusions, whereas apoptotic cells display characteristic shrinkage, intact plasma membranes, and apoptotic body formation [30]. Secondly, live-cell imaging techniques, including dual staining with propidium iodide (PI) and Annexin V, allow real-time monitoring of membrane integrity. Pyroptotic cells rapidly become PI-positive due to early pore formation, while apoptotic cells initially show Annexin V+/PI- staining (early apoptosis) progressing to Annexin V+/PI+ (late apoptosis). Lastly, beyond detecting pathway-specific biomarkers (e.g., GSDMD cleavage for pyroptosis vs. caspase-3 activation for apoptosis), rescue experiments using selective inhibitors (e.g., disulfiram inhibitors for pyroptosis or Z-VAD-FMK inhibitors for apoptosis) provide functional validation to distinguish these pathways [31].

These programmed cell death mechanisms have distinct differences and advantages. For pyroptosis, whether it is via a canonical, non-canonical, or alternative pathway, the common molecular mechanism involves cleaving GSDMs into N- and C-terminal fragments. According to current research, pyroptosis is characterized by chromatin fragmentation, an imbalance between intracellular and extracellular osmotic pressure, pore formation generated by the puncture of protease-cleaved GDSM proteins in the plasma or organelle membranes [3, 11, 32, 33], and discharge of cellular contents and proinflammatory cytokines that give rise to an inflammatory cascade reaction, such as IL-1β, IL-18, LDH, and high mobility group box 1 (HMGB1).

Contrary to previous conclusions, Boise et al. found that *Salmonella* induces cell death through a unique caspase-1-dependent mechanism rather than through an apoptotic pathway [34]. The phenomenon of pyroptosis was described by Zychlinsky et al. in 2001 in a study of macrophages infected with *Shigella flexneri*. The manner of cell death caused by bacterial infection and that caused by apoptosis are different; the former is referred to as pyroptosis [34, 35]. In 2002, Martinon et al. continued with similar research and found that a multiprotein complex was instrumental in activating caspase-1; they named this complex ‘inflammasome’[36, 37]. The discovery of an inflammasome protein complex was an enormous breakthrough contributing to the understanding of how the immune system triggers inflammation [37]. Inflammasome proteins are critical in the host defense against microbial pathogens [25]. Pyroptosis occurs through inflammatory caspases, which trigger the cleavage of the GSDM family and release of its C-terminal inhibitory and N-terminal effector domains [14]. Therefore, the roles of the GSDM and caspase protein families in the emergence of pyroptosis are essential. Inflammatory caspases (caspase-1, -4, -5, and -11) are also critical for innate immune defense. Caspase-1 is activated by ligands of various canonical inflammasomes, and caspase-11 directly acts as a receptor for cytosolic bacterial lipopolysaccharide (LPS) and is activated by binding to LPS [25], both of which trigger pyroptosis. The GSDM family is a protein family that executes cell death and comprises six members (GSDM A–E, and Pejvakin) in humans. Most GSDMs, except for DFNB59, have flexible linkages connected from the N-terminal pore-forming domain to the C-terminal inhibitory domain [7, 33]. Gasdermin D (GSDMD) is the substrate of proinflammatory caspases (caspase-1, -4, -5, and -11). Caspase-1 and caspase-4/5/11 can specifically cleave GSDMD, which is often in a state of self-inhibition that detaches the linkage between the amino-terminal GSDM-N and carboxy-terminal GSDM-C domains of GSDMD and liberates the pore-forming domain [33]. The purified N-terminal fragment, with intrinsic pyroptosis-inducing activity, moves to the plasma membrane during pyroptosis, efficiently lyses phosphoinositide/cardiolipin-containing liposomes, and perforates cell membranes [38]. The GSDMD protein is an executioner of pyroptosis [25, 39]. A major discovery was that the cleavage of GSDME by caspase-3 induces pyroptosis in certain GSDME-expressing cancer cells after chemotherapy, providing potential new approaches for cancer treatment [40]. In 2018, the Nomenclature Committee on Cell Death refined the definition of pyroptosis based on a previous concept, namely, a form of regulated cell death that critically depends on the formation of plasma membrane pores by members of the GSDM protein family, often as a consequence of inflammatory caspase activation [41]. Recently, pyroptosis has drawn increasing attention among researchers because of discoveries of its latest molecular mechanisms, and is increasingly regarded as a new direction for cancer immune therapies.

### Part 2: The activation pathway of pyroptosis

#### Molecules involved in pyroptosis

The formation of inflammasomes is a prerequisite for the activation of the canonical pathway of pyroptosis. Classical inflammasomes comprise the nucleotide-binding oligomerization domain-like receptor (NLR) family, pyrin domain-containing protein 1 (NLRP1), caspase-1, and apoptosis-associated speck-like protein (ASC); all are involved in caspase activation [36]. The initial response to infections caused by microorganisms is mediated by innate pattern recognition receptors (PRRs), which sense the presence of microorganisms and detect both exogenous pathogens, such as bacterial infections, and endogenous damage, including PAMPs and DAMPs [27, 42]. The NLR family, a subclass of PRRs, plays a pivotal role in initiating innate immune responses against cellular damage and stress signals [43]. Based on their N-terminal domain, NLRs are divided into five subfamilies: NLRA subfamily (acidic transactivating domain [AD]-containing NLR protein), NLRB subfamily (baculovirus inhibitor of apoptosis protein repeat [BIR]-containing NLR protein), NLRC subfamily (caspase activation and recruitment domain [CARD]-containing NLR protein, e.g., NLRC4, NLRC5), NLRP subfamily (pyrin domain [PYD]-containing NLR protein, e.g., NLRP3, NLRP1), and NLRX subfamily [44, 45]. Among these, NLRP1, NOD-like receptor protein 3 (NLRP3), and NOD-like receptor C4 (NLRC4) serve as the core sensor proteins of inflammasomes, driving caspase-1 activation and pyroptosis in response to distinct pathogenic or danger signals [46]. All members of the NLR family harbor a central NACHT domain (nucleotide-binding oligomerization domain shared by NAIP, CIITA, HET-E, and TP1) and a C-terminal leucine-rich repeat (LRR) domain [47]. Activation of PRRs by PAMPs or DAMPs contributes to the generation of inflammasomes. This process involves initiating the assembly of pro-caspase-1 via caspase activation and recruitment domains (CARD)–CARD interactions to recruit and ultimately boost caspase-1 dimerization and activation, which will also trigger downstream signaling cascades and lead to the production of type I interferon (interferon-α and interferon-β) and proinflammatory cytokines [14]. However, only PRRs containing CARD, such as NLRC4, can directly interact with pro-caspase-1 without the need for an adapter protein [48]. Certain PRRs with the pyrin domain (rather than CARD), such as NLRP3, can recruit pro-caspase-1 via ASC to form monomeric inflammasome units [49]. ASC serves as a bridge, which is attributed to the simultaneous structure of the pyrin and CARD domains [50]. One end of ASC combines with NLRP3, which is equipped with pyrin domains, and the other end binds the CARD domain of pro-caspase-1 [25]. In addition to the aforementioned NLRP3, NLRP1, and NLRC4, Absent in melanoma 2 (AIM2) is recognized as another core component of inflammasomes. Unlike NLR family sensors, AIM2 directly binds cytosolic double-stranded DNA (dsDNA) through its HIN200 domain, orchestrates ASC-dependent caspase-1 activation, and drives pyroptosis in response to viral infections or genomic instability [51, 52].

#### Canonical pathway

**Fig. 2:**
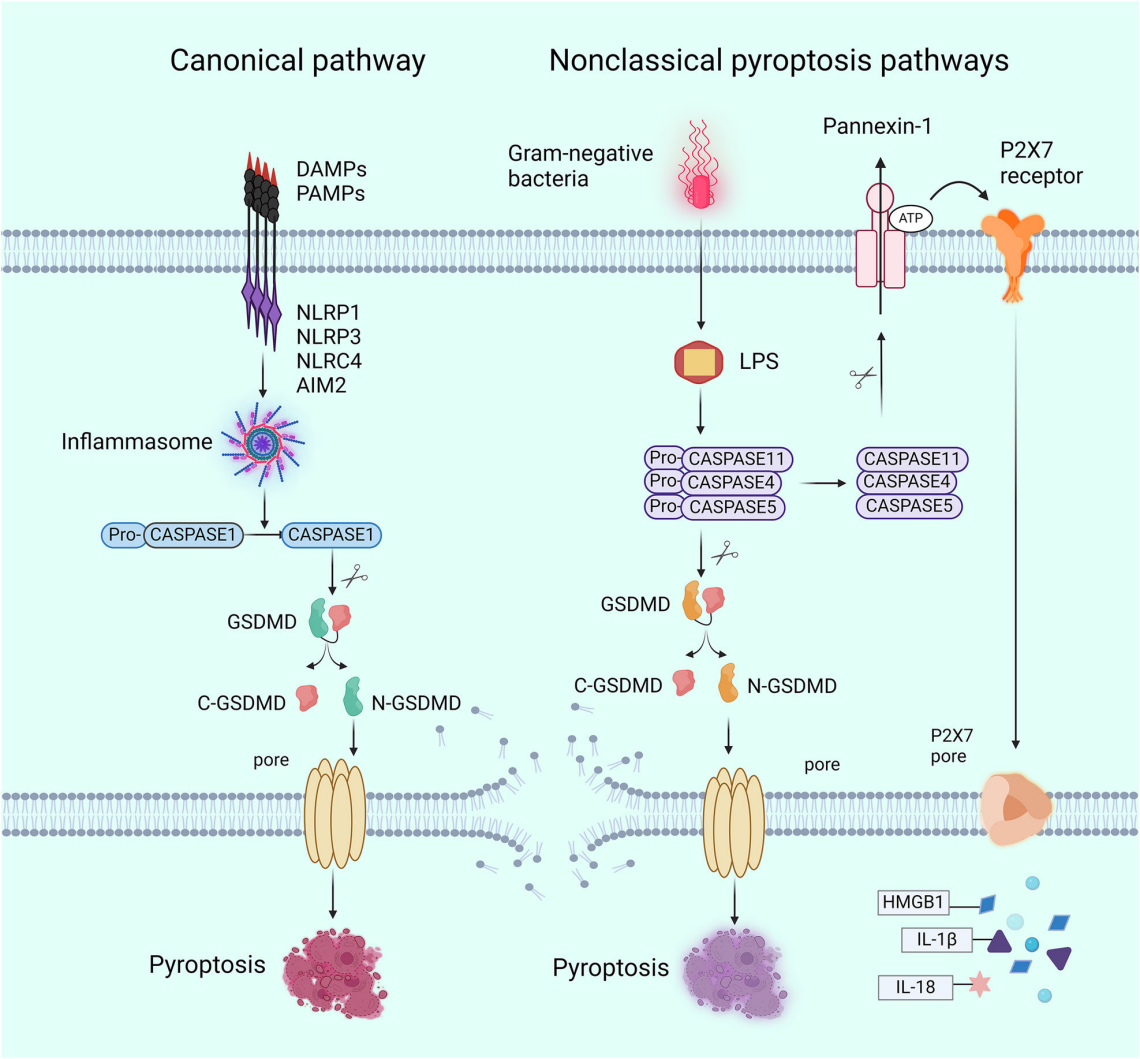
The classical and nonclassical pathways of pyroptosis. The pattern recognition receptors (PRRs) recognize DAMPs or PAMPs in the body, which activate inflammasomes and trigger the classic pathway of pyroptosis mediated by GSDMD. Intracellular LPS from gram-negative bacteria activates caspase-11, causing pyroptotic cell death. Caspase-4, -5 also directly recognize bacterial LPS to activate pyroptosis. In addition, activated caspase-11 can induce the cleavage of Panexin-1, leading to ATP and P2X7-mediated cell pyroptosis. DAMP danger-associated molecular patterns, PAMP pathogen-associated molecular patterns, GSDMD gasdermin D, LPS lipopolysaccharide; IL-1β interleukin-1β, IL-18 interleukin-18, HMGB1 high mobility group box 1 (created with BioRender.com) https://BioRender.com/9ag7zw9.

The canonical pathway is considered caspase-1-dependent. Pro-caspase-1 resides in the cytoplasm under normal circumstances in the form of the unreactive zymogen caspase-1. When recruited, pro-caspase 1 undergoes autocleavage to form active caspase-1 [13]. Caspase-1 can promote the maturation and secretion of proinflammatory cytokines (interleukin-1β [IL-1β] and IL-18) and can also trigger pyroptosis [12]. Caspase-1 can specifically cleave GSDMD, disrupting its hinge region. The N-terminal structure acts on the plasma membrane during pyroptosis and efficiently causes membrane permeabilization [38]. The net results are swelling and cell lysis (Fig. 2).

#### Nonclassical pyroptosis pathways

In addition to the canonical pathway, nonclassical pyroptotic pathways mediated by caspase-4/5/11 also exist [53]. Intracellular LPS from gram-negative bacteria activate caspase-11, causing pyroptotic cell death [54]. Caspase-4 and -5 also directly recognize bacterial LPS to activate pyroptosis and eliminate the need for inflammasomes [55]. Activated caspase-4/5/11 can also cleave GSDMD, which resembles the role of caspase-1, and results in the formation of cell membrane pores. Although caspase-1 is spared in the process of cleavage, caspase-1 and NLRP3 may be stimulated and activated to ultimately lead to the generation and emission of IL-18 and IL-1β. In addition, ATP release opens the membrane channel P2X7 via pannexin-1 activation mediated by caspase-4/5/11, creating a notch in the cell membrane and inducing pyroptosis [56, 57] (Fig. 2).

#### Alternative pathways

**Fig. 3:**
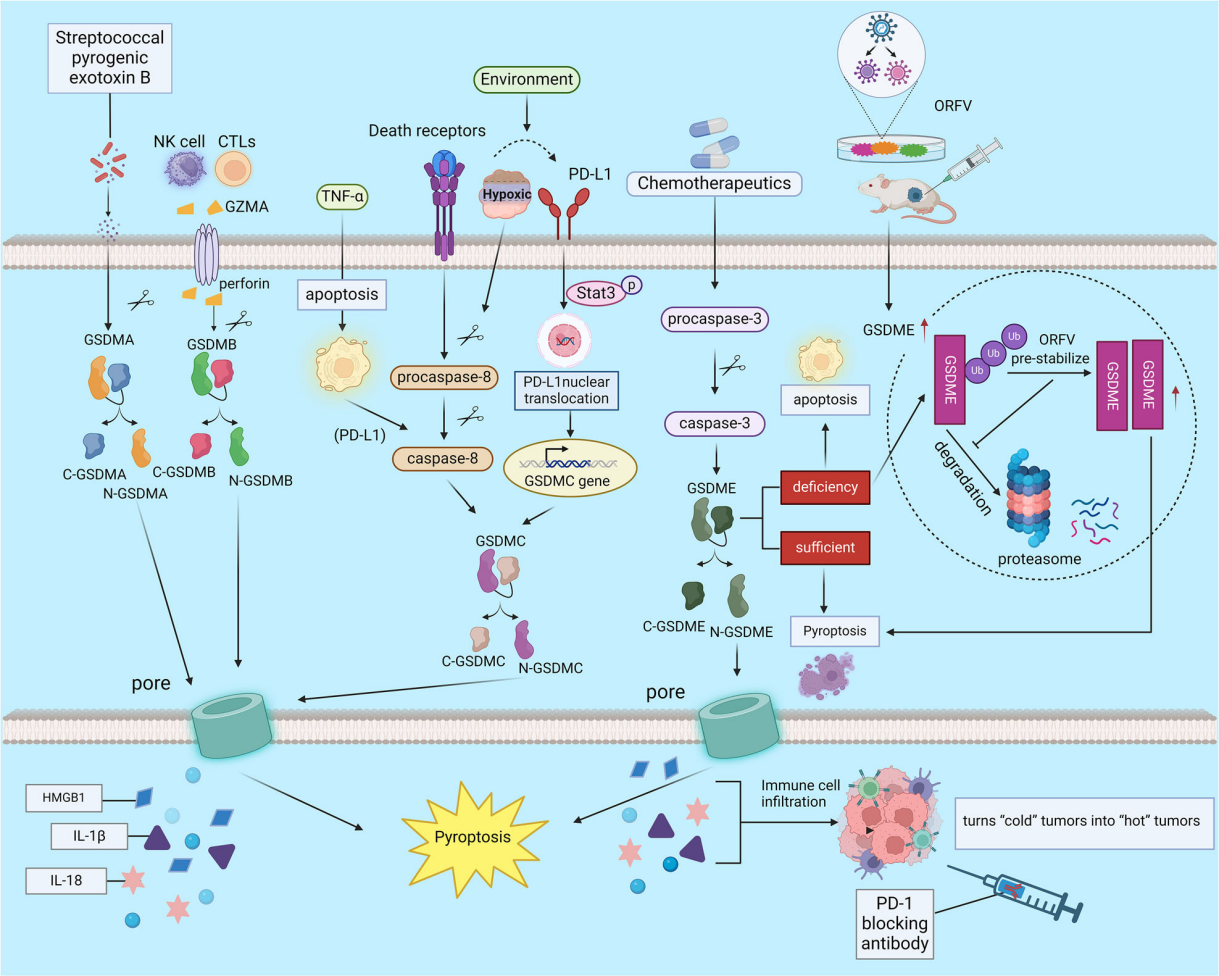
Alternative pathways for pyroptosis; these are mediated by members of the GSDM family rather than GSDMD. GSDM gasdermin, GSDMD gasdermin D (created with BioRender.com) https://BioRender.com/rircfrg.

Previously, investigators found that *streptococcal* pyrogenic exotoxin B cleaves GSDMA and induces pyroptosis [58]. This finding supplements that of other research regarding alternative pathways and underlines the latest progress in pyroptosis research. Granzymes, serine proteases secreted by cytotoxic T lymphocytes (CTLs) and natural killer (NK) cells, have been traditionally recognized for their role in eliminating target cells via activation of apoptotic pathways (e.g., caspase-3-dependent cleavage). However, recent studies have unveiled a non-canonical mechanism whereby granzymes—particularly Granzyme A (GZMA)—directly cleave GSDMB at the Lys229/Lys244 site, bypassing the classical inflammasome pathway to induce pyroptosis. This pathway operates independently of caspase-1/4/5/11 [59]. Hou et al. showed that PD-L1 has a considerable role during the transformation of TNFα-induced apoptosis into pyroptosis in cancer cells. Under hypoxia, p-Stat3 physically interacts with PD-L1 to boost its nuclear translocation. Hence, transcription of GSDMC increases [60]. Internalized death receptors are important for the assembly and activation of caspase-8. Death receptor 6 is a member of the death receptor family and is located upstream of caspase-8. One study suggested that death receptor 6 is an upstream factor of caspase-8 and GSDMC and may respond to the α-KG-induced increase in reactive oxygen species (ROS) signaling to induce pyroptosis [61]. Furthermore, activated caspase-8 cleaves GSDMC under hypoxia, liberating its N-terminal structure to induce membrane rupture [60]. Caspase-3 was originally identified as the core executor of apoptosis [62]. Current research shows that caspase-3 is involved in transforming apoptosis to pyroptosis by cleaving and activating GSDME after chemotherapy. TNF can also influence the transformation of apoptosis to pyroptosis through the caspase-3-GSDME axis [40]. A pivotal study reported that caspase 3/GSDME-mediated pyroptosis is highly dependent on the basal level of GSDME and that tumor cells lacking ‘sufficient’ GSDME undergo apoptosis instead of pyroptosis in the context of chemotherapy. However, Guan et al. showed that GSDME-low tumors can also trigger pyroptosis. These authors used oncolytic Orf virus (OrfV) to pre-stabilize GSDME by decreasing its ubiquitination [7]. Ultimately, the N-terminal domains (N-GSDME) are separated from full-length GSDME and transferred to the cell membrane, where they create pores [27, 39] (Fig. 3).

### Part 3: The relationship between pyroptosis, inflammation, and tumors

Pyroptosis is a highly inflammatory form of cell death; therefore, it can arouse the immune response, remodeling the TME and turning immunologically ‘cold’ tumors into ‘hot’ tumors more easily. Initially, pyroptosis was found to be involved in the immune defense against infections [3]. Under conditions of various infections and immunological challenges, activated canonical caspase-1 and different inflammasomes participate in pyroptosis, and GSDM proteins have emerged as core protagonists in the inflammatory response against pathogens [27, 63]. Pyroptosis and inflammasomes are responsible for the processing of inflammatory cytokines and the stimulation of inflammatory cascades. However, excessive inflammasome activation results in inflammatory damage, including damage that leads to chronic inflammation-related cancers [64]. Therefore, a clear understanding of inflammasomes and pyroptosis is vital for ameliorating inflammatory diseases. GSDM proteins originally exist in full-length and inactive forms [27]. Under the inducement of diverse stimuli and the activation by various cellular signals, GSDM proteins are subjected to proteolytic cleavage, and the oligomerized N-terminal domain of GSDM proteins is allowed to penetrate the cell membrane and cause rapid plasma membrane rupture, thus releasing proinflammatory mediators such as IL-18 and IL-1β [33, 65]. The proinflammatory effect of IL-1β/IL-18 and pyroptosis could contribute to the development of autoimmune and inflammatory diseases and recruit immune cells to the site encountering infection [25]. These proinflammatory mediators have also been found to promote tumor progression [66]. Moderate pyroptosis defends against pathogens by eliciting an inflammatory response. Conversely, undue and excessive pyroptosis may be damaging and exacerbate sepsis and septic shock [67]. Inflammasomes and the microenvironment of tumor cells are associated with the development of cancer [2]. If genetic damage is the “match that lights the fire” of cancer, some types of inflammation may provide the “fuel that feeds the flames” [68]. Thus, inflammation and cancer have a close affinity. Long-term exposure of tissues and/or cells to an inflammatory environment increases cancer risk [13]. We hypothesize that the enhanced inflammatory microenvironment caused by pyroptosis may facilitate the development and proliferation of tumors. However, the elicitation of pyroptosis enhances immune activity by upregulating the infiltration of CD8^+^ T cells, NK cells, and M1 macrophages [27]. Pyroptosis may affect immunosuppression and facilitate systemic immune responses in solid tumors [69].

**Fig. 4:**
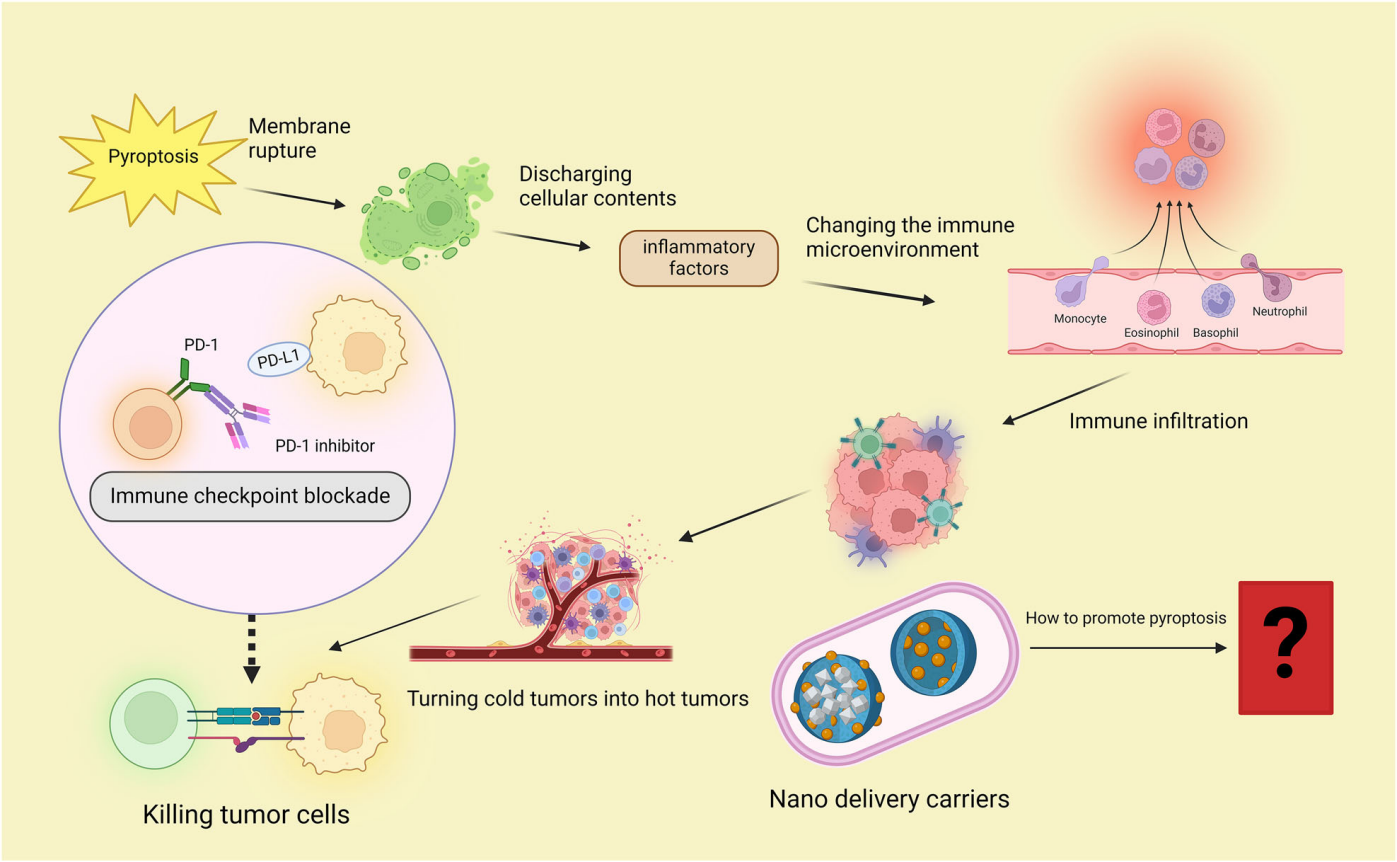
Relationship between pyroptosis, inflammation, and tumors. (Created with BioRender.com) https://BioRender.com/lhwohy1.

Pyroptosis, as a pro-inflammatory programmed cell death mechanism, demonstrates significant therapeutic advantages worth leveraging, particularly in its capacity to activate anti-tumor immunity. However, careful consideration must be given to the potential complications arising from excessive inflammatory responses. The intricate regulatory mechanisms underlying pyroptosis necessitate precise modulation to achieve an optimal balance between maximizing therapeutic efficacy and minimizing adverse effects. For instance, nanotechnology and ultrasound-targeted strategies, such as the hydralazine-loaded nanodroplets (HYD-NDs) nanosystem developed by Li Jie’s team, exemplify this approach. This pH- and ultrasound-responsive platform enables tumor microenvironment-specific drug release, achieving precise induction of pyroptosis through localized upregulation of GSDME expression and caspase-3 activation. This dual-stimuli-responsive design ensures spatiotemporal control over pyroptotic cell death, effectively minimizing off-target toxicity while amplifying immunogenic antitumor responses [70]. The auxiliary role of nanomaterials in biomedical applications is gradually becoming prominent. Furthermore, combination therapeutic strategies amplify synergistic effects through multi-modal interventions. Promising approaches include: Integration with immune checkpoint inhibitors and pyroptosis-released DAMPs enhances dendritic cell maturation and CD8⁺ T cell infiltration, converting immunologically “cold” tumors into “hot” microenvironments to potentiate PD-1/PD-L1 blockade efficacy [7]. Pharmacological modulation of tumor metabolic reprogramming (e.g., metformin-mediated activation of AMP-activated protein kinase [AMPK]) synergizes with pyroptosis induction through disruption of energy homeostasis in malignant cells [71]. Thus, a logical relationship between pyroptosis, inflammation, and cancer has been confirmed. Pyroptosis can trigger an inflammatory response, which is equivalent to immune activation. Tumor immunity and immunotherapy are associated with immune activation (Fig. 4).

After tumor cells undergo pyroptosis, the cell membrane ruptures and the contents of the cells are released, including some inflammatory factors, which changes the immune microenvironment around the tumor cells, increases immune cell infiltration, and transforms ‘cold’ tumors into ‘hot’ tumors. Thus, using immune checkpoint blockade (ICB) therapy to eradicate tumor cells will be more effective. Finally, the important role of nanomaterial delivery systems in triggering pyroptosis cannot be underestimated.

### Part 4: Relationship of pyroptosis in tumorigenesis

Pyroptosis-related genes are closely associated with multiple types of tumorigenesis; therefore, the specific mechanism of pyroptosis and its role in tumorigenesis deserve further study.

#### Pyroptosis in lung cancer

Based on previous studies on human cancers, researchers have found that GSDMB is highly expressed in both healthy and cancerous tissues [1]. Inflammasome proteins involved with pyroptosis, such as NLRP3 and AIM2, contribute to tumorigenesis by modulating immunity and crosstalk between the microenvironment and lung epithelial cells [3]. Globally, lung adenocarcinoma (LUAD) is the leading cause of death. Tumors driven by oncogenic KRAS account for ~30% of LUAD cases. Currently, effective chemotherapy for such tumors is lacking [72]. Cisplatin is a chemotherapeutic agent that is used to treat lung cancer [73]. Accumulating evidence indicates that pyroptosis contributes to the reversal of chemotherapeutic resistance in lung cancer. Recently, Cui et al. discovered that patients with LUAD with a high expression of pyroptosis-related genes were more sensitive to cisplatin and had a better prognosis [74]. Furthermore, the authors demonstrated that combining inetetamab and cisplatin could trigger NLRP3/caspase-1/GSDMB-mediated pyroptosis to bolster anti-LUAD efficacy by curbing HER2/AKT/Nrf2 signaling to increase ROS levels. GSDMC is also highly expressed in LUAD and may be a promising predictive factor for poor prognosis [75]. GSDMD levels are dramatically increased in non-small cell lung carcinoma (NSCLC) [76]. Gao et al. showed that increased expression of GSDMD significantly correlated with the aggressiveness of NSCLC, and that the TNM stage was higher and the tumor was larger in patients with NSCLC with increased expression of GSDMD than in those without increased expression [76]. Another study found that GSDMD expression was closely associated with CD8^+^ T cell markers in the Cancer Genome Atlas cohorts. GSDMD cleavage increased in CTLs and human-activated CD8^+^ T cells. The authors also highlighted the requirement for GSDMD to obtain an optimal CTL response to cancer cells [77]. GSDME expression differs in multiple tumors. Recent studies have confirmed that caspase-3 and GSDME can work as a “switch” to transform apoptosis to pyroptosis in GSDME-high expression cells [3, 40]. Regarding reinforcing NSCLC, GSDME enhances cisplatin sensitivity by mediating pyroptosis to trigger antitumor immunocyte infiltration. The discovery of the immunological regulatory effects of GSDME on lung cancer provides a new potential target to enhance lung cancer immunotherapies [78]. These findings highlight the potential immunotherapeutic implications of targeting GSDME to reduce chemotherapy resistance in the treatment of lung cancer. An interesting phenomenon has also been observed in lung cancer cells treated with paclitaxel and cisplatin. Cisplatin induced higher levels of secondary necrosis/pyroptosis than paclitaxel in A549 cells, suggesting that cisplatin may provide additional advantages in treating lung cancers with high levels of GSDME expression [73]. Recently, the role of GSDME in the pathogenesis of human malignancies has attracted considerable attention. The occurrence of pyroptosis due to chemotherapy is worth further research and consideration to elucidate unexpected effects and the potential of overcoming chemotherapy resistance.

#### Pyroptosis in gastric cancer

Gastric cancer is the fifth most common type of cancer and the third leading cause of cancer-related deaths worldwide, causing more than 720,000 deaths per year due to its malignant nature [79, 80]. Recently, Komiyama et al. detected GSDMB expression in cancerous stomach tissue samples and discovered that GSDMB is widely expressed in gastric cancer [81]. Saeki et al. [82] identified an enhancer region in GSDMB that actuates gene expression in gastric cells and found that two promoters, a cellular promoter and a long terminal repeat-derived promoter, are capable of driving GSDMB expression. Additionally, the long terminal repeat promoter drives GSDMB expression in gastric cancer specimens. Yu et al. investigated the relationship between duodenogastric reflux and the onset of gastric cancer. They found that macrophages upregulated the expression of ubiquitin-specific protease 50 in both active human bile reflux gastritis and murine duodenogastric reflux models. Ubiquitin-specific protease 50 accelerates bile acid-induced NLRP3 inflammasome activation and pyroptosis by interacting with deubiquitinating ASC in macrophages. The release of HMGB1 facilitated by pyroptosis begets gastric tumorigenesis by PI3K/AKT and MAPK/ERK pathways [64]. Yin et al. investigated the role of pyroptosis-related genes in gastric cancer and found that GSDME closely correlated with immune checkpoints in gastric cancer. GSDME expression is accompanied by intensified phagocytosis of tumor cells by tumor-associated macrophages, and the number and function of tumor-infiltrating NK cells and CD8^+^ T cells also increase. Activated caspase-3 cleaves GSDME, resulting in its N-terminal fragment. Most pyroptosis-related genes are upregulated in gastric cancer [83].

#### Pyroptosis in colon cancer

Xie et al. revealed that simvastatin triggers pyroptosis via the ROS/NLRP3/caspase-1/GSDMD pathway in colon cancer. This finding provides novel insights into future understanding of the mechanism of action of simvastatin in colon cancer treatment [84]. Recently, IL-17A (a proinflammatory cytokine primarily secreted by Th17 cells, γδT cells and NK cells) was found to induce mitochondrial dysfunction and pyroptosis through the ROS/NLRP3/caspase-4/GSDMD pathway and promote intracellular ROS accumulation. In addition, IL-17A can foster the secretion of inflammatory factors, such as IL-1β, IL-18 and immune antigens, and recruit CD8^+^ T cells to infiltrate tumors [85]. Guan et al. generated different ORFV recombinants with one or two gene deletions and found that WT ORFV and ORFV recombinants triggered GSDME-mediated pyroptosis in human colon cancer tissues ex vivo. ORFV-triggered GSDME-mediated tumor pyroptosis recruits CTLs to the TME. The infiltration and activation of CTLs within tumor lesions result in more effective responses to immune checkpoint inhibitors [7]. Zhang et al. demonstrated that coxsackie virus group B3 facilitated significant tumor regression by cleaving GSDME but not GSDMD. Coxsackie virus group B3 exerts oncolytic activity in colon cancer cell lines via GSDME-mediated pyroptosis [86]. Oncolytic viruses have the beneficial ability to exert a dual effect of directly eliminating tumors while facilitating an anti-tumor immune response [7].

#### Pyroptosis in ovarian cancer

Although ovarian cancer is less common than cervical and breast cancer, the 5-year survival rate is less than 50%; ovarian cancer has more malignant attributes and a worse prognosis. With the popularity of immunotherapy in cancer biology, combining targeted therapy and immunotherapy is a promising strategy for ovarian cancer treatment. Pyroptosis has also been shown to promote immunotherapy in ovarian cancer, including treatment with chimeric antigen receptor T cells or immune checkpoint inhibitors. Pyroptosis in ovarian cancer is also affected by inflammasomes, various signaling pathways, and long non-coding RNA [87]. A result shows that SF3B1 is shown to be overexpressed and related to low cytotoxic immune cell infiltration in ovarian cancer. Targeting SF3B1 reprograms the immunosuppressive tumor microenvironment in ovarian cancer and synergizes with ICB. Pladienolide B, an SF3B1 inhibitor, increases the expression of PD-L1 [88]. Combining an SF3B1 inhibitor and ICB to treat ovarian cancer may be an ideal treatment. Yang et al. showed that CBL0137 also has an anti-tumor role in ovarian cancer cells in vivo. CBL0137 can inactivate the chromatin remodeling complex, generating a transcriptional decrease in chromatin and antioxidant genes and inducing caspase-3/GSDME-dependent pyroptosis via the ROS/BAX pathway [89]. These findings may broaden the scope for the treatment of ovarian cancer by expanding the approach to pyroptosis.

#### Pyroptosis in glioma

Glioma, the predominant form of central nervous system malignancy, is highly prevalent and has a low 5-year survival rate [90]. Studies have indicated that several regulatory non-coding RNAs can induce pyroptosis in glioma tumor cells and restrain their proliferation in vitro [91]. TREM2, a pyroptosis-related regulator of glioma, was found to strengthen tumor cell proliferation and invasion. One study suggested that inflammation and necrosis may boost the migration and invasion of glioma stem cells [92]. Therefore, the authors speculated that the intricate microenvironment caused by pyroptosis may result in the conversion and upregulation of oncogenes, thereby provoking tumor proliferation [91, 93].

### Part 5: Application of nanomedicine delivery combined with pyroptosis in the fight against cancer

Pyroptosis is closely associated with inflammation and immunity, even in the TME, because of its proinflammatory form. ICB therapy has proven clinical benefits for multiple cancers, including the liberation of tumor antigens, stimulation of valid tumor immunogenicity, and improvement of the efficacy of ICB [94]. However, effective pyroptosis for treating tumors is limited. Biodegradable pyroptosis inducers for safe and effective treatment of tumors are scarce and have drawbacks such as insolubility, high removal rate, and non-specific distribution [95]. To overcome these limitations, many efforts have been made to develop nanomaterial-based drug delivery systems for nanozymes. Nanomaterials can serve as a carrier or agency and provide tremendous superiority in targeted delivery for expediting anti-tumor therapy. With rapid advancements in nanotechnology, stimuli-responsive nanomaterials have emerged as feasible choices for designing controlled drug delivery systems [96]. The advent of numerous approaches for encapsulating bioactive ingredients in nanodelivery systems has improved the stability and targeted delivery of biomolecules [97]. The combination of nanomaterials and immunotherapy has shown promise in completely eliminating tumors owing to excellent anti-tumor effects and negligible side effects [98]. Several studies have found that different nanozymes or nanomaterials can be applied to induce pyroptosis, which could eventually lead to more promising treatments for cancer.

#### Hollow carbon nanozymes

Tao et al. developed a mild hyperthermia-enhanced pyroptosis-mediated immunotherapy platform utilizing iron- and copper-decorated hollow carbon nanozymes. These engineered nanostructures, exhibiting multi-enzyme mimetic activities, were designed to induce pyroptotic cell death through the ROS-Tom20-Bax-Caspase3-GSDME signaling pathway upon light activation. Experimental results demonstrated the superior efficacy of these bimetallic carbon nanozymes in triggering pyroptosis across both in vitro and in vivo tumor models. Furthermore, the combination of photothermally activated pyroptosis with anti-PD-1 checkpoint blockade therapy showed synergistic enhancement of antitumor immunity, suggesting a promising combinatorial approach for cancer immunotherapy [94].

#### Phthalocyanine-conjugated mesoporous silicate nanoparticles

Zhang et al. developed phthalocyanine-conjugated mesoporous silicate nanoparticles (PMSN) for sonodynamic therapy (SDT). These engineered nanostructures enhance therapeutic efficacy by amplifying oxidative stress through dual mechanisms: their porous architecture doubles ROS generation under ultrasound activation, and the induced cavitation effect potentiates pyroptotic cell death within tumor tissues. Systematic evaluations confirmed that PMSN-mediated SDT significantly expands pyroptotic cell populations via ROS overload [99].

#### Proteolysis-targeting chimeras

Proteolysis-targeting chimeras (PROTAC) have shown promise for inducing post-translational knockdown of target proteins in disease treatment. Bromodomain-containing protein 4 (BRD4) is a nuclear protein essential for gene transcription and can be degraded by PROTAC. A nano “targeting chimera” (L@NBMZ) consisting of BRD4-PROTAC combined with a photosensitizer has been developed. L@NBMZ blocks gene transcription by degrading BRD4 through proteasomes in vivo and induces the cleavage of caspase-3. Photosensitizers facilitate the cleavage of caspase-3 and its pyroptosis [100]. This study employs nanoscale engineering to enhance tumor-targeting specificity and simultaneously uncovers a synergistic antitumor mechanism involving pyroptosis pathway activation and epigenetic regulation.

#### Metal-organic frameworks

Targeted drug delivery systems based on metal-organic frameworks (MOFs) have progressed tremendously since their inception and are now widely applicable in diverse scientific fields [101]. MOFs, a unique class of synthetic polymeric materials formed through coordination between metal species and organic ligands, are characterized by structural customizability [102]. Consequently, the integration of MOF with nanomaterials has been extensively investigated. In an in vivo study, Zhen et al. designed Hf-TBP/cholesterol oxidase (COD), a COD-functionalized nanoscale MOF, to achieve cholesterol depletion and mechanical regulation of tumors. Hf-TBP/COD induces calcium ion influx, inhibits cell migration, enhances rupture propensity for effective caspase-1-mediated pyroptosis, and reduces oxidative stress tolerance. Additionally, Hf-TBP/COD increases the mechanical tension of plasma membranes and the osmotic fragility of cancer cells. Within the TME, Hf-TBP/COD downregulates multiple immunosuppressive checkpoints to reinvigorate T cells and promote their infiltration [103].

Chen et al. synthesized a zeolitic imidazolate framework-8 (ZIF-8) nanoparticle loaded with hydrophobic chlorin e6 (Ce6) and hydrophilic tirapazamine (TPZ), further modified with a gastric cancer cell membrane to enhance targeting specificity. ZIF-8 is an MOF synthesized using Zn^2+^ ions and 1,2-methylimidazolate. Ce6 can be easily loaded onto ZIF-8 via strong electrostatic interactions between the Ce6 and Zn^2+^ ions. The pH-responsive ZIF-8 structure releases Ce6 in the acidic TME. Upon ultrasound irradiation, activated Ce6 generates ROS to directly kill tumor cells. Concurrently, the hypoxic TME potentiates TPZ activation, amplifying cytotoxicity and synergizing with chemotherapy and SDT. ROS further act as NLRP3 inflammasome activators, promoting caspase-1 activation through the NLRP3 inflammasome pathway. This cascade ultimately cleaves gasdermin D (GSDMD), inducing pyroptosis in gastric cancer cells [104].

#### Nanogels

Nanogels are aqueous dispersions of submicron-sized, three-dimensional, strongly cross-linked networks of hydrophilic polymers that have been studied as drug delivery systems owing to their biocompatibility, high stability, flexible particle size, and drug loading [105]. Zhang et al. executed glutathione-responsive nanogels (referred to as IMs), which are composed of crosslinker DBHD with the BRAF inhibitor dabrafenib and the COX2 inhibitor celecoxib. These nanogels are able to effectively induce tumor cell pyroptosis to exert robust antitumor immunity. In addition, glutathione-responsive nanogels combined with αPD-1 antibody primarily suppressed tumor growth and extended survival time in a melanoma mouse model [106]. Xu et al. developed a glutathione/ROS dual-responsive nanogel system capable of actively targeting the overexpressed mannose receptors (MR) on cancer cells while enhancing the photothermal efficacy of indocyanine green (ICG). Upon light irradiation, photoactivated ICG induces cytoplasmic Ca^2+^ influx and activates caspase-3. This cascade triggers pyroptosis and augments the tumor immune response through NLRP3 inflammasome activation. [107]. Balahura et al. constructed a composite polysaccharide hydrogel using cellulose nanofibers (CNFs) and alginate/pectin (A.CNF or P.CNF). Furthermore, the composite polysaccharide hydrogel effectively delivered 5-fluorouracil (5-FU). P.CNF/5-FU scaffolds can inhibit breast tumor cell growth and induce inflammasome complex activation, together with extra- and intracellular ROS generation to trigger pyroptosis [108].

#### Liposomes

Liposomes are bilayer or multilayer lipid vesicles, in which the aqueous volume is entirely enclosed by a membrane composed of amphiphilic phospholipids. The polar end (hydrophilic) faces the external aqueous environment, whereas the non-polar end (hydrophobic) forms a hydrophobic environment. This structure allows liposomes to deliver drugs with different physicochemical properties. Among these nanocarriers, liposomes are one of the most successful candidates for delivering targeted oncological treatment, improving the safety profile and therapeutic efficacy of encapsulated drugs [109]. Zhong et al. constructed a nanoliposome (GM@LR) for co-delivering the GSDME-expressing plasmid and manganese carbonyl (MnCO) into triple-negative breast cancer cells. The MnCO generated Mn^2+^ and carbon monoxide (CO) in the presence of H_2_O_2_. The CO-activated caspase-3 cleaves GSDME, thereby converting apoptosis to pyroptosis in 4T1 cells. Mn^2+^ promotes the maturation of dendritic cells by activating the STING signaling pathway. The increased proportion of mature intratumoral dendritic cells results in a massive infiltration of CTLs, leading to a robust immune response [110]. Zhang et al. developed cisplatin-loaded nanoliposomes to investigate epigenetics-driven tumor cell pyroptosis, aiming to enhance the immunomodulatory efficacy of chemotherapeutic nanocarriers. When combined with the DNA methyltransferase inhibitor decitabine, cisplatin synergistically restored GSDME protein expression, thereby inducing pyroptosis in colon cancer cells [111].

The aforementioned material shows that the involvement of nanomaterials and immunotherapy has infinite potential to improve the treatment of cancer by triggering pyroptosis.

## Conclusion and perspectives

Pyroptosis has the potential to increase options for confronting cancer owing to its preponderance of immunity-related capacity. The effects of pyroptosis could be significant in the quest to eradicate cancer. The prospects of combining nanodelivery with targeted pyroptosis therapy are vast. In the field of tumor treatment, this combination therapy has the potential to maximize treatment effectiveness and significantly reduce toxic side effects.

Although the delivery system of nanomaterials has improved, certain challenges remain unaddressed. Exploring treatments with better tumor-targeting performance, devising multiple targeted medicines with more precise functions, resolving the issue of the lack of tumor retention capacity, and improving the most effective delivery systems of nanodrugs are imperative actions required to improve the efficacy of cancer therapy.

## Data Availability

No datasets were generated in researching and writing this review.
